# Insoluble (1 → 3), (1 → 4)-β-D-glucan is a component of cell walls in brown algae (Phaeophyceae) and is masked by alginates in tissues

**DOI:** 10.1038/s41598-017-03081-5

**Published:** 2017-06-06

**Authors:** Armando A. Salmeán, Delphine Duffieux, Jesper Harholt, Fen Qin, Gurvan Michel, Mirjam Czjzek, William G. T. Willats, Cécile Hervé

**Affiliations:** 1 Department of Plant and Environmental Sciences, University of Copenhagen, Thorvaldsensvej 40, 1871 Frederiksberg, Denmark; 2 Sorbonne Universités, UPMC Univ Paris 06, UMR 8227, Integrative Biology of Marine Models, Station Biologique de Roscoff, CS 90074 Roscoff, France; 3 CNRS, UMR 8227, Integrative Biology of Marine Models, Station Biologique de Roscoff, CS 90074 Roscoff, France; 4 Carlsberg Research Laboratory, J.C. Jacobsens Gade 4, 1799 København V, Denmark; 5 William G.T. Willats, Newcastle University, Newcastle upon Tyne, United Kingdom

## Abstract

Brown algae are photosynthetic multicellular marine organisms. They belong to the phylum of Stramenopiles, which are not closely related to land plants and green algae. Brown algae share common evolutionary features with other photosynthetic and multicellular organisms, including a carbohydrate-rich cell-wall. Brown algal cell walls are composed predominantly of the polyanionic polysaccharides alginates and fucose-containing sulfated polysaccharides. These polymers are prevalent over neutral and crystalline components, which are believed to be mostly, if not exclusively, cellulose. In an attempt to better understand brown algal cell walls, we performed an extensive glycan array analysis of a wide range of brown algal species. Here we provide the first demonstration that mixed-linkage (1 → 3), (1 → 4)-β-d-glucan (MLG) is common in brown algal cell walls. Ultra-Performance Liquid Chromatography analyses indicate that MLG in brown algae solely consists of trisaccharide units of contiguous (1 → 4)-β-linked glucose residues joined by (1 → 3)-β-linkages. This regular conformation may allow long stretches of the molecule to align and to form well-structured microfibrils. At the tissue level, immunofluorescence studies indicate that MLG epitopes in brown algae are unmasked by a pre-treatment with alginate lyases to remove alginates. These findings are further discussed in terms of the origin and evolution of MLG in the Stramenopile lineage.

## Introduction

Brown algae are a large and diverse class of marine and photoautotrophic organisms, consisting of more than 250 genera and 1500–2000 species^1^. They form the monophyletic class of Phaeophyceae which is included in the Stramenopile lineage and that forms one of the eight major phylogenetic groups of eukaryotes^2^. Thus, brown algae evolved complex multicellularity independently from animals, fungi, red algae, green algae and green plants^2, 3^. The Stramenopiles also comprise the pseudofungi oomycetes, the photosynthetic unicellular diatoms (Bacillariophyceae) and various unicellular or filamentous algae, including the Eustigmatophyceae and Xantophyceae which are more closely related to brown algae^4, 5^. Brown algae have an important ecological impact as they are the largest biomass producers in coastal regions. Their environment is very different to that of land plants, being submerged in salt water and exposed to a changing environment of current, waves and tides, to which they adjust quickly to comply with bending or torsion forces^6^.

As in other multicellular photosynthetic organisms, brown algae evolved a carbohydrate-rich cell-wall surrounding their cells. However, due to the aforementioned phylogenetic distances with other lineages, their cell wall composition and characteristic are distinct^7^. The two main polysaccharides are gel-forming and hydroscopic polymers, namely the alginates and the fucose-containing sulfated polysaccharides (FCSPs), the latest including sulfated fucans^8^. The cellulose microfibrils are believed to be cross-linked by the FCSPs and to have no connection with alginates^8^, which in turn are associated with phenolic compounds to form a network in which the elements cited above are embedded. Additional cell wall components are proteins, arabinogalactan proteins (AGPs) and halide compounds^8, 9^. Cell wall rigidity is likely controlled by the tuning of alginate fine structure and polyphenol cross-linking, while sulfated polysaccharides may play a key role in the adaptation to osmotic stress^8, 10^.

In brown algal cell walls the polyanionic polysaccharides are prevalent over neutral and crystalline polysaccharides. The later only represent a minor fraction of the wall (~0–10%) and are believed to be composed mostly, if not exclusively, of cellulose^11^. More recent studies point out that some brown algae are able to build up (1 → 3)-β-glucan chains in their wall, transiently upon infection in some Ectocarpales and Laminariales^12^, or more ubiquitously in *Fucus vesiculosus* cell walls^13^. To the best of our knowledge no additional neutral and/or water insoluble polysaccharide has been reported so far in brown algal cell walls. The presence of an unbranched and unsubstituted glucan featuring a mixture of (1 → 3)-β- and (1 → 4)-β-d-linked glucose residues, also known as a mixed linkage glucan (MLG), has been suspected but never clearly determined^14^. The presence of MLG has been however established in the stramenopile xantophyte alga *Monodus subterraneus* ^15^.

MLG has a peculiar distribution pattern among eukaryotes, being mostly found in the plant kingdom and in fungi^16^ and with additional occurrences reported in red algae^17^ and bacteria^18, 19^. MLG in the green lineage is mostly distributed in the members of the Poaceae, but also in some less commonly found early diverging vascular plants and freshwater green algae^20–23^. The fact that MLG occurs in some of these evolutionarily very distant taxa but not in all, congruent with some genetic evidences, is in favour of a hypothesis based on multiple origins of the polymer, being ‘reinvented’ several times even at the level of the plant kingdom^24^. In plants the ratio between the two linkages seems to be species specific and to influence the physicochemical properties of the polysaccharide and consequently its functional properties in cell walls^25^.

In an attempt to better understand brown algal cell walls, particularly in term of their glucan composition, we performed an extensive glycan array analysis of a wide range of brown algal species, included within the most important phaeophycean orders. This led to the discovery that MLG epitopes are ubiquitous to all the phylogenetically diverse brown algal cell walls tested in this study. We describe here the highly regular structure of MLG in brown algae, and its association with alginates in tissues. We further discuss the origin and evolution of MLG in this eukaryotic lineage.

## Results

### Discovery of MLG in cell walls of brown algae

**Figure 1:**
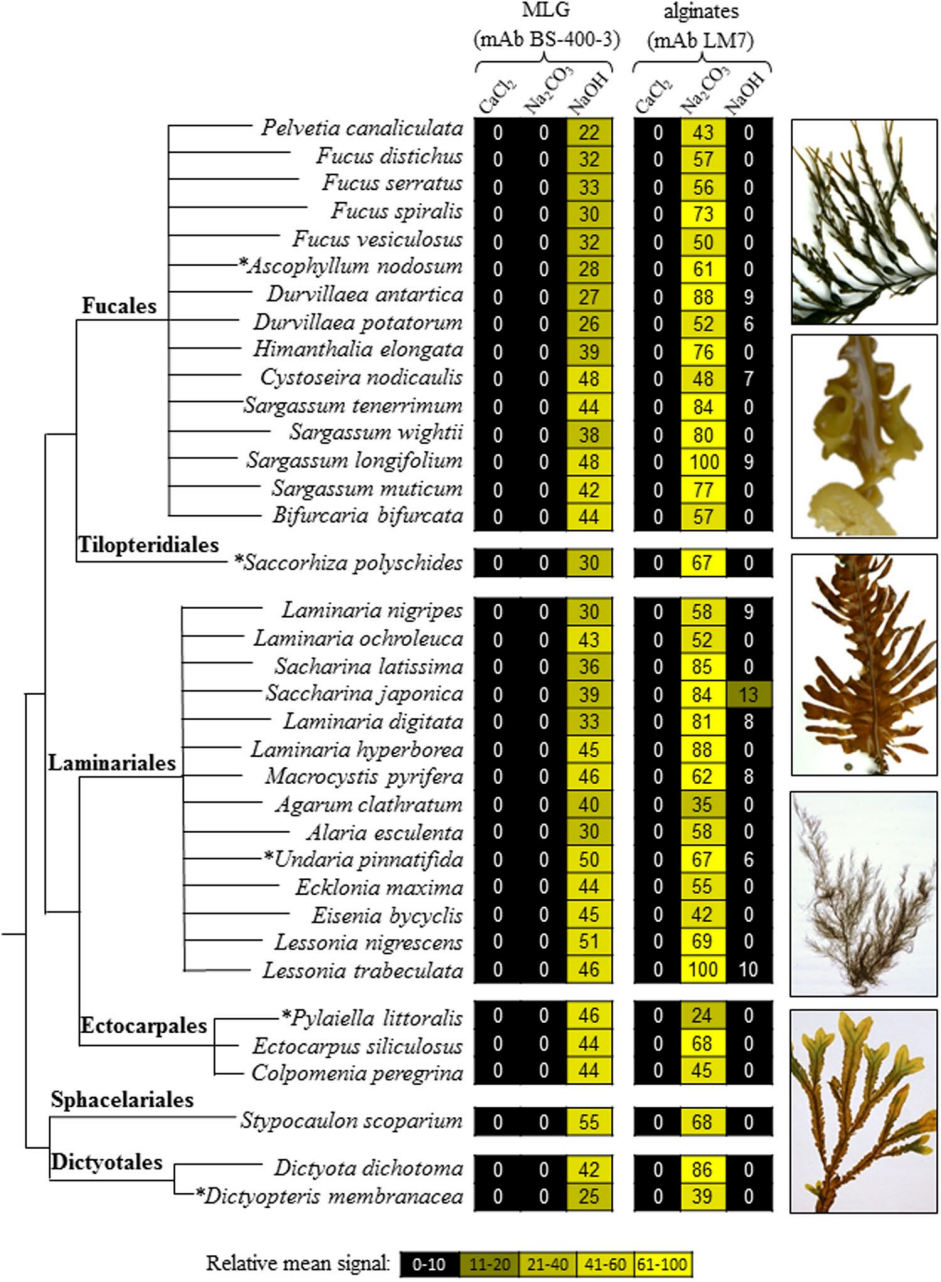
Detection of MLG epitopes in brown algal extracts by glycan array analyses. The tree shows the schematic phylogenetic relationships of the 34 species of brown algae used in the study: they belong to 6 main orders of brown algae from the early diverging clade Dictyotales to the more recently diversified clades of Laminariales, Fucales and Ectocarpales. Cell walls were sequentially extracted and probed with the anti-mixed linkage glucan (MLG) and anti-alginates (LM7) antibodies. The heatmap represents the binding profile obtained from the glycan array analyses. The colour scale in relation to absorbance values is shown. For each antibody the highest mean signal value in the entire data set was set to 100 and all other signals adjusted accordingly. Values <5 were considered as background and discarded. Stars by algal names refer to the corresponding pictures shown on the side.

Cell walls from a range of brown algal species of distinct taxonomical groups were sequentially extracted using a dedicated protocol. Three solvents (CaCl_2_, Na_2_CO_3_, NaOH) were chosen because they are known to preferentially solubilise FCSPs, alginates and water insoluble polysaccharides (such as cellulose), respectively^10, 13^. The presence of MLG in these fractions was challenged by antibody-based glycan array analyses. The monoclonal antibody LM7 is directed to pectic homogalacturonan but also binds to alginates^10^, and was used as a positive control. As expected, alginates were abundantly detected in the Na_2_CO_3_ fractions but not in the NaOH fractions. Unexpectedly, MLG was readily detectable in all the brown algal species under investigation (Fig. 1). The MLG occurrence was restricted to the NaOH-fractions, which contain components held firmly in cell walls. In some brown algal species, further analysis of cell wall extracts taken from blade, stipe or holdfast regions indicated that the detected amounts of MLG were similar between tissues (Supplementary Information Fig. S1).

**Figure 2:**
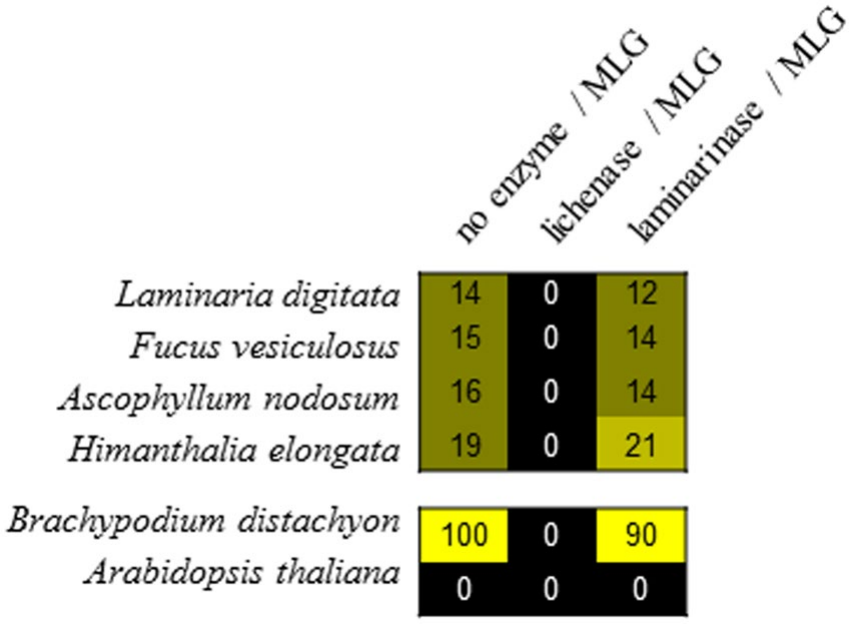
Deletion of MLG epitopes from glycan arrays by enzymatic treatment. Glycan arrays were treated with buffer only or with either a lichenase (10 µg/ml) or a laminarinase (7.4 µg/ml) for 5 min before probing with the anti-mixed linkage glucan (MLG) and anti-alginates (LM7) antibodies. *Brachypodium dystachion* is included as a positive control, while *Arabidopsis thaliana* is included as negative control.

In order to rule out any possibility that the antibodies were cross-reacting with other polymers than MLG in this context, and to validate the presence of MLG in brown algal cell walls, we used a specific enzymatic-based epitope deletion assay (Fig. 2). MLG can be hydrolysed specifically by a lichenase, an MLG-specific endohydrolase whose target site is known to be a (1 → 4)-β-d-glucopyranosyl linkage immediately adjacent to a (1 → 3)-β-d-glucopyranosyl residue^16^. It does not hydrolyse pure (1 → 3)-β-d-glucans such as laminarin (e.g. the main storage polysaccharide in brown algae) nor (1 → 4)-β-d-glucans (cellulose). A dedicated array printed with the NaOH fractions of several brown algal samples was prepared and submitted to lichenase digestion before probing. As a control a parallel analysis was performed on *Brachypodium distachyon* (Poaceae) which is known to have the highest content of MLG among grasses^25^ and on *Arabidopsis thaliana* (Brassicaceae) which does not contain MLG. As expected MLG was abundantly detected in the *B*. *distachyon* extract but was not detected in *A*. *thaliana*. MLG was present in the brown algal extracts but detected at lower levels as compared to *B*. *distachyon*. In all samples the MLG signal was abolished by the lichenase treatment. An additional control used a laminarinase that preferentially cleaves soluble (1 → 3)-β-d-glucans such as laminarin^26^. MLG-epitopes in brown algae were unaffected by this treatment. A reduction of the MLG signal in *B*. *distachyon* is likely attributed to the lichenase side-activity of the laminarinase^26^, on a prevailing and more readily accessible MLG substrate in these cell wall extracts as compared to those from brown algae. Altogether these results indicate that the anti-MLG antibody is not cross-reacting with any putative remaining laminarin in this context and that the polysaccharide detected in the brown algal extracts is a (1 → 3), (1 → 4)-β-d-glucan sensitive to a lichenase treatment.

### MLG in brown algae has a highly regular structure

**Figure 3:**
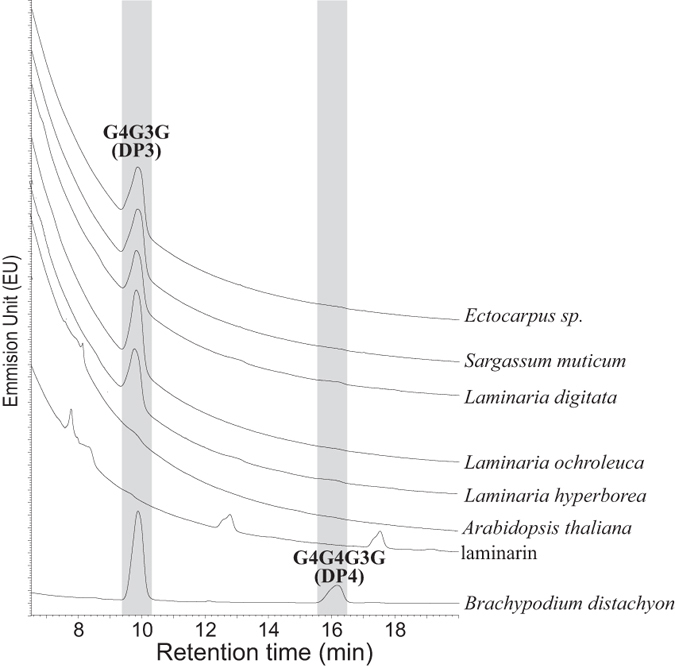
UPLC analysis of lichenase oligomers. Ultra-Performance Liquid Chromatography (UPLC) analysis of the oligosaccharide products released by the lichenase digestion of cell wall extracts obtained from 6 brown algae belonging to 3 distinct orders (Ectocarpales, Fucales, Laminariales). *Brachypodium dystachion* is included as a positive control, while *Arabidopsis thaliana* and a commercial laminarin sample are included as negative controls. The chromatogram shows the relative abundance of the tri- (G4G3G) and tetra-saccharides (G4G4G3G). Note that the algal MLG has a very regular structure with trisaccharides only, in contrast to *B*. *dystachion* which has both tri- and tetrasaccharide units.

By cleaving solely the (1 → 4)-β-D-glucopyranosyl linkages immediately adjacent to (1 → 3)-β-D-glucopyranosyl residues, the digestion of MLG by the lichenase yields diagnostic oligosaccharides. For instance, the major oligosaccharides released by lichenase digestion of poalean MLG are the trisaccharide G4G3G (DP3) followed by the tetrasaccharide G4G4G3G (DP4)^25^, where ‘G’ is β-d-glucopyranose, and ‘3’ and ‘4’ indicate (1 → 3) and (1 → 4) linkages. To better characterise the MLG structure in brown algae, AIRs were digested with the lichenase. The oligosaccharides released from the lichenase digestion were fluorescently labelled and analysed with an Ultra-Performance Liquid Chromatography (UPLC) coupled to a fluorescence detector (Fig. 3). No overlap between starch and cellulose derived oligosaccharides DP3 and DP4 (maltotriose, maltotetraose, cellotriose, cellotetraose) was observed (Supplementary Information Fig. S2). Five algal samples corresponding to three different orders (Ectocarpales, Fucales and Laminariales) were assayed. In addition to the *B*. *distachyon* and *A*. *thaliana* extracts, a commercial laminarin sample was included as a negative control. Consistent with previous analyses, the most abundant hexose oligomers in *B*. *distachyon* MLG were the DP3 (G4G3G) followed by the DP4 (G4G4G3G)^25^. *A*. *thaliana* was negative as it does not possess MLG. Some peaks were observed from the laminarin sample but none overlapping with the DP3 and DP4 from MLG. As expected, the lichenase has no activity towards laminarin. This experiment rules out that the eluted oligosaccharides are derived from a lichenase activity and indicates that they were initially present in the laminarin sample. By contrast, all the five algal samples under investigation offer a similar pattern of elution to the one observed for *B*. *distachyon* with however some quantitative distinctions. As anticipated from the glycan array analyses, the amounts detected were very low compared to the positive control. More importantly, only the DP3 was detected in reasonable quantities, indicating that the MLG in brown algae has a very regular block structure consisting of cellotriose units linked by single (1 → 3)-β-d-linkages (G4G3G). We noted the presence of some faint signal at the MLG derived DP4 level in some of the samples, and we cannot rule out that some irregularities in the linkage pattern might be present in some individuals, species or life-cycle stages.

### Immunolocalisation of MLG in brown algal tissues

**Figure 4:**
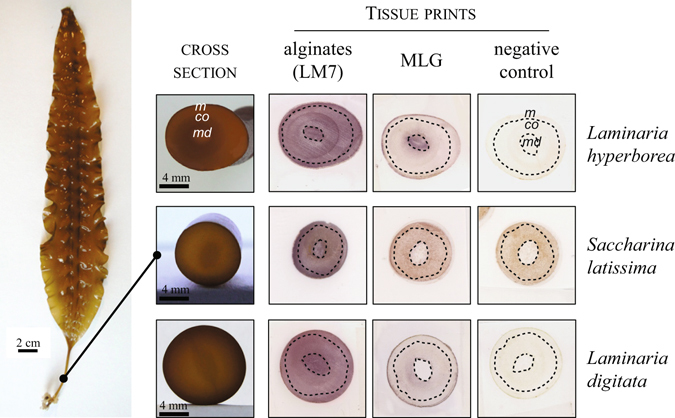
Detection of MLG in stipes from Laminariales by tissue printing. The tissue prints of stipes from Laminariales were probed by the anti-mixed linkage glucan (MLG) and anti-alginates (LM7) antibodies. The samples being naturally pigmented, negative controls are included to appreciate the signal due to the antibody binding. Corresponding cross sections of the stipes are shown on the side. A representative picture of a whole plant of *Saccharina latissima* is indicated for size indication. The LM7 epitopes are abundant in all tissues. MLG is of a more restricted occurrence and shows distinct spatial localization depending on the alga. It is mostly detected in the medulla for *Laminaria hyperborea*, while it is more largely distributed in the meristoderm and the cortex in *S*. *latissima*. m, meristoderm; co, cortex; md, medulla. The dot lines broadly indicate the different regions in tissues as observed by light microscopy.

Based on our understanding that MLG is an insoluble polymer firmly wall-bound in brown algae we hypothesised that it might contribute to the stiffness of some of the tissues. We explored its location in the stipes of Laminariale species, a stem-like rigid structure that connects the flexible body blade to the anchoring holdfast. Cut surfaces of stipes were tissue-printed and the nitrocellulose sheets were immunolabelled to detect cell-wall epitopes. The positive control LM7 reacted strongly with the tissue prints which indicates the abundant and consistent distribution of alginates within the stipes (Fig. 4). Much of the spatial information regarding distribution is maintained during the printing process and indicated that the LM7 labelling was stronger in the radial rows separating the distinct tissue layers, and in the medulla of *L*. *hyperborea* and *L*. *digitata*. The presence of MLG within the stipes was also supported by a positive immunolabelling of the prints. MLG showed a more restricted occurrence as compared to alginates, being detected in all meristoderms, but showing distinct spatial localisations depending on species for other tissues. It was mostly detected in the medulla for *L*. *hyperborea*, while it was more largely distributed in the cortex for *S*. *latissima* and *L*. *digitata*. It is interesting to mention that tissue printing usually allows the transfer of soluble material, indicating that the insoluble MLG may have been dragged along with other polysaccharides which it could be associated to, and alginates would be a possible candidate.

**Figure 5:**
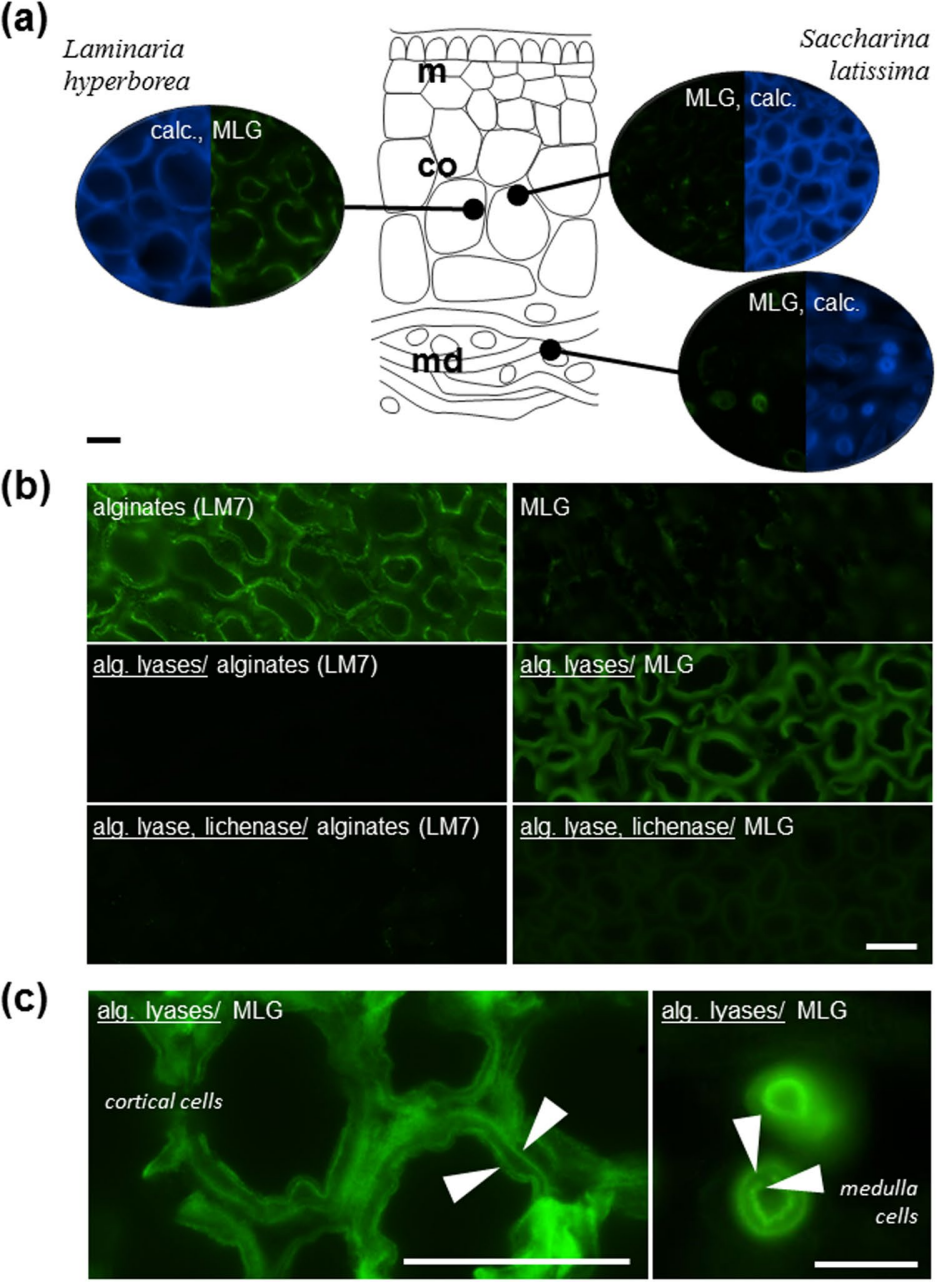
Unmasking MLG in stipes from Laminariales by immunofluorescence imaging. (**a**) Overview of MLG detection in cross-sections of stipes from *Laminaria hyperborea* and *Saccharina latissima*. The diagram illustrates the distinct tissues under study. The micrographs show the indirect immunofluorescence detection of MLG epitopes for some of the tissues. The Calcofluor White (calc.) stains all cell walls in sections. MLG is not or only weakly detected. The strongest binding is obtained in the cortical cell walls from *Laminaria hyperborea*. (**b**) Indirect immunofluorescence detection in cortical cell walls from *Saccharina latissima*. MLG epitopes are strongly detected in all cell walls after a pre-treatment with alginate lyases. Equivalent sections are labelled with LM7 and indicates the loss of alginate detection after the enzymatic treatment. Equivalent sections further treated with a lichenase indicate a decrease in MLG detection. (**c**) Same sections observed at higher magnification indicate that the MLG epitopes are strongly detected after the enzymatic treatment in the most inner and outer parts of the cell walls, both in cortical and medulla cells. m, meristoderm; co, cortex; md, medulla. All scale bars = 50 μm.

To study in more details the MLG distribution in these tissues, we cross-sectioned stipes from these Laminariale species and localised MLG by immunofluorescence microscopy (Fig.5, Supplementary Information Fig. S3). Our first attempts to localise MLG in these tissues were disappointing as almost no signal was obtained, or only at a very low level for *L*. *hyperborea* (Fig. 5a). Knowing that in plants pectic homogalacturonans can mask the detection of hemicellulose polysaccharides^27, 28^, and that MLG is probably associated to alginates in our samples, we performed an unmasking experiment based on the use of recombinant alginate lyases from marine bacteria^29, 30^. The treatment was effective in removing most alginates as shown by the loss of LM7 binding (Fig. 5b). After degradation of alginates, MLG was clearly labelled in all cell walls and particularly in the cortex of *S*. *latissima* where it appears to be more easily labeled (Fig. 5b). The signal was abolished by an additional lichenase treatment, indicating that the antibody was not cross-reacting with other polymers in this context but truly detecting MLG. Observations at higher magnifications indicated that MLG had a restricted distribution to the most inner and outer part of the cell wall (Fig. 5c).

## Discussion

Cell walls of brown algae have been extensively studied in term of composition, notably regarding their gel-forming polysaccharides alginates and sulfated fucans/FCSPs^8^. Cellulose is the only crystalline component which has been reported in the walls from brown algae so far and it only occurs at low levels^11^. In eukaryotes MLG has been largely studied in the green lineage where it has been adopted by different plant and algal species during the course of evolution^20–23^, and in fungi where it is known as lichenan^16, 31, 32^. The discovery of MLG in brown algae was therefore unexpected. Our survey revealed that MLG epitopes are not only present but ubiquitous to all brown algal cell walls tested in this study. Most studies on brown algal cell walls have been focusing on the economically relevant polymers and the water insoluble residues have therefore been only poorly described. It could be argued that glucose residues measured within these fractions were assumed to derive from cellulose. Some rare and old studies already depicted the presence of a mixture of (1 → 3)-β- and (1 → 4)-β-linked glucose residues in alkali-soluble materials of *Fucus vesiculosus* ^33, 34^ but the chemical structure of the originated polymer was not established. More recent studies point out that some brown algae are able to build up (1 → 3)-β-d-glucan chains in their wall, transiently upon infection in some Ectocarpales and Laminariales^12^, or more ubiquitously in *Fucus vesiculosus* cell walls^13^. The presence of the two linkage types on the same glucan chain was already suspected but not conclusively determined^14^. This is the first report which uses a combination of chemical and enzymatic analyses and that demonstrates the presence of the two adjacent linkage types as a form of an insoluble MLG in brown algal cell walls.

MLG of various eukaryotic and bacterial origins have been structurally resolved. The use of a specific (1 → 3), (1 → 4)-β-d-glucan endohydrolase, known as a lichenase, has been particularly important in this matter. The lichenase hydrolyses a (1 → 4)-β-d-glucopyranosyl linkage immediately adjacent to a (1 → 3)-β-d-glucopyranosyl residue. Digestion of MLG either yields a trisaccharide (G4G3G) followed by a tetrasaccharide (G4G4G3G) in cereal grains^16, 25^, an asymmetry which is even more pronounced in fungi^32^. The reverse is observed in the early diverging land plants *Equisetum spp*. with a predominance of tetrasaccharides over di- and tri-saccharides^21, 23, 35^, and a combination of these two profiles can be observed in green algal species^36^. Additional structural variations include the consecutive linkage alternation in the acetylated MLG from the bacteria *Sinorhizobium meliloti* ^19^ and the occurrence of a sulfated MLG in the red alga *Kappaphycus alvarezii* which contained only a minor (~8% mol) fraction of (1 → 3)-β-linkages^17^. Brown algal MLG is rather unique in structure in containing almost exclusively the trisaccharide (G4G3G) as a repeating unit. This regular conformation may allow long stretches of the molecule to align and therefore to form well-structured micofibrils insoluble in water: an analysis that fits with the observation of MLG being only extracted by high alkali in our brown algal samples.

MLG in brown algae is a component of a cell wall architecture that drastically differs from what is known in other lineages such as land plants and fungi. Instead of co-exisiting with high levels of either glucuronoarabinoxylans (e.g. Poales), pectins and cellulose (e.g. *Equisetum spp*.) or (1 → 3), (1 → 6)-β-d-glucans (e.g. Ascomycota), MLG occurs in brown algae with high levels of alginates and sulfated fucans/FCSPs, together with low levels of cellulose. In all brown algae, MLG was left in the residue after sequential extraction with CaCl_2_ and Na_2_CO_3_, being only extracted with high alkali, suggesting that it is firmly wall-bond and co-extracted with cellulose microfibrils. Our findings also seem to indicate that MLG may be associated with another cell wall polymer. In cell wall immunochemistry studies, we hypothesized that it might be alginates, since the binding of the anti-MLG antibody to untreated transverse sections of stipes of Laminariales indicated a weak recognition of highly restricted locations and binding was shown to be increased considerably by the enzymatic removal of alginates. The detection of both MLG and alginates in the tissue prints also indicated that MLG might have been dragged along with other polysaccharides. On the other hand, however, the glycan array analysis indicates a contradictory result, as no MLG was detected in the fractions based on the use of Na_2_CO_3_, which is effective in releasing most (but not all) alginates. All together, the presented results here do not yet allow any conclusion on the potential crosslinking of MLG structures with other polysaccharides.

The conformational regularity of the (1 → 3), (1 → 4)-β-linkages will define the MLG tendencies to self-aggregate^16^. This points to a putative major structural role for MLG in brown algal cell walls. This regular glucan block structure also reflects a precise biosynthetic mechanism and/or an important functional requirement in brown algal cell walls. MLG in cereals differ in the peculiar distribution of the linkages along the chain, which has a profound influence on the rheological properties of the polysaccharide, thus forming a gel-like matrix in cell walls^16^. In the grasses, MLG accumulates transiently in young and growing tissues therefore it has traditionally been associated with the regulation of cell wall expansion^37^. In *Equisetum spp*. it is believed to serve a different purpose and has been suggested to be involved in silica deposition^21^. Brown algal cell walls are not known to contain silica and our findings show that MLG has no distinction of occurrence between the tissue-types but is consistently present at low levels. These results suggest that MLG may perform a distinct role in brown algae compared to land plant organisms, probably in strengthening the cell wall.

The non-conserved structural conformation of MLG between lineages, as its uneven distribution pattern among eukaryotes, is indicative of an independent evolution of MLG synthesis in multiple species. Candidate genes encoding cellulose synthase-like (Csl) proteins from the glycosyltransferase GT2 family, have been identified that might participate in MLG synthesis in land plants. The synthesis of MLG in the Poaceae is effective by the evolution of a single clade of genes, namely CslF or CslH genes^16^. Recent progress has been made in unravelling the mechanism of MLG synthesis in plants, with the identification of specific amino acid changes in CslF6 isoforms that can influence the proportions of the different repeating units^38, 39^. How a single protein can generate the different block structures with two linkage-types is yet still a matter of debate^16, 25^. None of these genes has been found in any other MLG-producing plants and green algae and it has therefore been postulated that other genes are responsible for producing MLG in these organisms^24^. Also consistent with this, no CslF or CslH homologues were found in the genome sequence of the brown algal model *Ectocarpu*s *siliculosus* ^40, 41^, and the nine GT2 genes identified in *E*. *siliculosus* form two distinct GT2 clades^41^. Interestingly some of these genes are more closely related to *BcsA* genes from bacteria than *Csl* genes from plants^42^, while the bacterial *BscsA* gene has recently been shown to be involved in MLG synthesis in *Sinorhizobium meliloti* ^19^. The *E*. *siliculosus* GT2 genes which are more closely related to the *BcsA* genes would therefore be serious candidates for the production of MLG in brown algae.

Major changes in cell wall composition often accompanied key evolutionary events, such as territorialisation of charophytes and their descendants, namely land plants^7, 43^. In the stramenopile brown algae the recruitment of new cell wall materials such as polyphenols and alginates, is believed to have shaped the building of complex multicellularity^8, 41^. The recruitment of insoluble and tightly assembled MLG-chains would have helped in providing structural rigidity for the development of semi up-right forms. However, to fully understand cell wall evolution in the lineage, a comprehensive investigation of cell wall across all stramenopile groups would be needed. So far our understanding of cell wall structures is limited to the major groups (i.e. brown algae, oomycetes), with only a fragmentary view for other groups that evolved in-between. Alginates are known to be produced by a restricted number of stramenopile taxas basal to brown algae (i.e. Schizocladiophyceae, Xantophyceae) but in a cell wall architecture primarily made of cellulose in the case of the xantophyte *Monodus subterraneus* ^15^. Interestingly, MLG has also been shown to be present in this latter organism, with 15% of (1 → 3)-β-linkages, but it is not known if the block structure resembles the brown algal repeat-unit^15^. In the earlier group of the Eustigmatophyceae, MLG production has not been reported but some GT2 genes in *Nannochloropsis gaditana* are related to those of the brown alga *E*. *siliculosus* and to the bacterial *BscsA* genes^42^. Further studies including less-well studied species would be therefore valuable to address whether or not the biosynthetic mechanisms of MLG biosynthesis are deeply conserved within the stramenopiles, or rather involve non-related sets of glycosyl transferases. These findings would also help in defining the main cell wall modifications that arose during the course of evolution in the Stramenopile lineage.

## Methods

### Collection of algal samples

All samples of algae, except the *Ectocarpus siliculosus* sample, were collected from natural environments. These samples were collected from Roscoff (France, GPS coordinates: 48.72, −3.98), unless otherwise stated: *Dictyopteris membranacea*, *Dictyota dichotoma* (Dictyotales); *Colpomenia peregrina*, *Pylaiella littoralis*, *Ectocarpus sp*. (Ectocarpales); *Fucus vesiculosus* from Aarhus (Denmark, 56.16, 0.20), *F*. *spiralis* from Aarhus, *F*. *serratus* from Aarhus, *F*. *distichus* from Aarhus, *Ascophyllum nodosum* from Grenaa (Denmark, 56.42, 10.88), *Himanthalia elongata* from Plougerneau (France, 48.62, −4.56), *Pelvetia canaliculata* from Plougerneau, *Durvillaea antartica* from Chiloé-Dalcahue (Chile, 42.60, −73.95), *Durvillaea potatorum* from Girvan (UK, 55.24, −4.86), *Sargassum wightii* from Chennai (India, 13.00, 80.32), *S*. *tenerrimum* from Chennai, *S*. *muticum*, *S*. *longifolium* from Chennai, *Cystoseira nodicaulis*, *Bifurcaria bifurcata*, (Fucales); *Stypocaulon scoparium* (Sphacelariales); *Saccorhiza polyschides* from Galway (Ireland, 53.00, −9.55) (Tilopteridales); *Macrocystis pyrifera* from Chiloé-Dalcahue, *Laminaria ochroleuca* from Plougerneau, *L*. *nigripes* from Godthåb/Nuuk (Greenland, 64.18, 51.72), *L*. *hyperborea* from Plougerneau, *L*. *digitata* from Plougerneau, *Saccharina japonica* from Chiba (Japan, 36.61, 140.12), *S*. *latissima* from Godthåb/Nuuk, *Lessonia trabeculata* from Chiloé-Dalcahue, *Le*. *Nigrescens* from Chiloé-Dalcahue, *Eisenia bicyclis* from Chiba, *Ecklonia maxima* from Cape Town (South Africa, −34.31, 18.46), *Undaria pinnatifida*, *Agarum clathratum* from Godthåb/Nuuk, *Alaria esculenta* from Godthåb/Nuuk (Laminariales). Macroalgae were cleaned of epiphytes and washed in tap water. No permissions were required for these locations/activities. The collection of algal samples did not involve endangered or protected species. *Ectocarpus siliculosus* (Ec32 strain), was cultured in Station Biologique de Roscoff (France) as described previously^10^.

### Plant material

*Brachypodium distachyon* and *Arabidopsis thaliana* were cultivated in greenhouse conditions at the University of Copenhagen. Seedlings of *Brachypodium distachyon* (L.) P. Beauv. line 21 (Bd21) were grown at average temperatures of 22/16 °C (day/night) at ambient light levels. The mature plants were kindly provided by Vanja Tanackovic Fangel. Seedlings of *Arabidopsis thaliana* Columbia line (Lehle Seeds, USA) were grown in hydroponics at average temperatures of 24/17 °C (day/night) at ambient light levels. Rosettes and roots were collected from 12-week old plants. The collected plant materials were freeze-dried before extractions.

### Cell wall extractions and microarray profiling

The cell-wall extracts were prepared as previously described^9^. In short, after preparation of the insoluble alcohol-residues (AIRs), the cell-wall polymers were sequentially extracted using a dedicated protocol for brown algae based on the subsequent use of 2% CaCl_2_, 3% Na_2_CO3 and 4 M NaOH. Two intermediate treatments with 0.2 M HCl and 50 mM CDTA respectively, were used to ensure a correct enrichment of fractions (Supplementary Information Fig. S1). Samples were centrifuged between each extraction (1500 g, 15 min) and the supernatants collected. Samples of *Brachypodium dystachion* and *Arabidopsis thaliana* were treated following the same protocol and used as a positive and a negative control, respectively. Extracts were then printed on nitrocellulose membranes using a microarrayer robot (piezoelectric Sprint Arrayjet, Roslin, UK) in four dilutions and four technical replicates. The microarrays were probed and developed as described previously^44^ using the following antibodies: anti-MLG from Biosupplies (BS-400-3) and LM7 from Plant Probes (anti-homogalacturonan cross-reacting and detecting alginates^10^). These probed arrays were scanned and signals were quantified using the Array Pro-Analyzer 6.3 software (Media Cybernetics, Rockville, MD, USA) to be ultimately converted into heatmaps, in which mean spot signals of the 16-spot sub-arrays are correlated to colour intensity. For each algal sample the data are based on three repeats of the whole process.

In some cases the microarrays were treated with enzymes prior to antibody labelling (epitope deletion). Degradation of soluble (1 → 3)-β-d-glucans was performed using the laminarinase LamA from *Zobellia galactanivorans* ^26^ at a concentration of 7.4 µg/ml. Degradation of MLG was performed using a (1 → 3), (1 → 4)-β-d-glucanase known as a lichenase from *Bacillus sp*. (Megazyme) at a concentration of 10 µg/mL. The two enzymes were applied independently to the microarrays in 100 mM Tris pH6.5, 200 mM NaCl for 5 min at room temperature. Arrays not treated with the enzymes were incubated for an equivalent time with the same buffer. All arrays were subsequently treated for 45 min with 10 µg/ml proteinase K (Sigma-Aldrich) in PBS before probing to remove any enzyme still attached to the cell wall extracts. The arrays were washed again with PBS before probing.

### Chromatographic separation

Six brown algae including one Ectocarpale (*Ectocarpus sp*.), one Fucale (*Sargassum muticum*) and three Laminariales (*L*. *digitata*, *L*. *ochroleuca*, *L*. *hyperborea*) were harvested in Roscoff, cleaned of epiphytes and dried. After preparation of the AIRs the cell wall components were extracted with 4 M NaOH. For each algal species, cell wall were extracted from a bulked mixture of two to four individuals to account for natural biological variations. The supernatants were neutralized with acetic acid until pH 6.5 and a (1 → 3), (1 → 4)-β-d-glucanase treatment was applied for 2 hours using the lichenase from *Bacillus sp*. (Megazyme) at a concentration of 3.3 U/mg of sample in 10 mM phosphate buffer pH6.5. The resulting oligomers were fluorescently labelled with 2-aminobenzamide and analysed with a Waters Acquity Ultra-Performance Liquid Chromatography (UPLC) System equipped with an Acquity glycan column and a fluorescence FLR detector (excitation wavelength of 350 nm and emission wavelength of 420 nm), using an Acquity UPLC BEH glycan 1.7 μm, 2.1 × 150 mm column with a VanGuard BEH glycan 1.7 μm, 2.1 × 5 mm pre-column at room temperature. Samples of *Brachypodium dystachion* and *Arabidopsis thaliana* were treated following the same protocol and with the same enzyme and used as one positive and one negative control, respectively. The *Brachypodium* sample is presented compressing the scale by eight times for the purpose of visualization. An additional negative control using ethanol-washed commercial laminarin (Sigma); that naturally contains shorter, purely β-1,3-linked gluco-oligosaccharides) was treated with the same lichenase for 2 hours at a concentration of 3.3 U/mg of sample in 10 mM phosphate buffer pH6.5. For each sample and for each algal species, at least four technical replicates of the chromatograms were produced. In order to eliminate possible co-elution between the MLG trisaccharide, cellotriose or maltotriose, appropriate controls consisting of MLG (Glucagel) (DKSH, Italy), maltotriose, maltotetraose, cellotriose, cellotetraose (all Sigma, USA) were treated as described above. No co-elution could be observed between the MLG trisaccharide and any of the standards (Supplementary Information Fig. S2).

### Tissue printing

Stipes of Laminariales (*S*. *latissima*, *L*. *hyperborea*, *L*. *digitata*) were cross-sectioned and pressed firmly onto a nitrocellulose sheet. Biological replicates of the sections for each stipe were produced from two seasonally distinct individuals (collected in April and October). The most representative section was then chosen to be displayed. The prints were allowed to dry for 1 h and then blocked with 3% (w/v) milk powder in PBS (PBS/MP) for 1 h. The antibodies targeting MLG (BS-400-3, Biosupplies) and alginates (LM7, Plant Probes) were added at 10-fold and 1000-fold dilutions respectively, in PBS/MP and incubated for 1 h. After washing, the secondary antibodies linked to alkaline phosphatase (AP) were added at 1000-fold and 5000-fold dilutions in PBS/MP (e.g. anti-mouse for anti-MLG and anti-rat for LM7, respectively). After a further 1 h the nitrocellulose sheets were washed in water and the binding detected by addition of AP substrate (450 µM of 5-Bromo-4-chloro-3-indolyl phosphate disodium, 400 µM Nitrotetrazolium Blue chloride). Negative controls were treated in the same manner as described, except that no MLG or alginate specific antibodies were added at any point of the experiment. The reaction was stopped by washing extensively in water.

### Immunomicroscopy

Stipes of Laminariales (*S*. *latissima*, *L*. *hyperborea*, *L*. *digitata*) were sectioned at 60 µm thickness using a Leitz 1320 freezing microtome (Ernst Leitz Wetzlar GmbH, Wetzlar, Germany) and immediately fixed overnight in 4% paraformaldehyde in 50% diluted seawater. For the immunolabelling procedure, the samples were washed twice in PBS and incubated in PBS/MP for 1 h. Samples were then washed in PBS and the antibodies targeting MLG (BS-400-3, Biosupplies) and alginates (LM7, Plant Probes) were added at 10-fold and 1000-fold dilutions, respectively, in PBS/MP and incubated for 1 h. Following washing with PBS, the secondary antibodies linked to fluorescein isothiocyanate (FITC) were added at 100-fold dilutions in PBS/MP in darkness (e.g. anti-mouse for anti-MLG and anti-rat for LM7; Sigma). After 1 h, the samples were washed with PBS, incubated with Calcofluor White (Sigma) for 5 min in darkness and mounted after washing in a glycerol-based anti-fade solution (Citifluor AF31; Agar Scientific). All slides were observed on an Olympus BX60 microscope equipped with epifluorescence irradiation. Images were captured with an Exi Aqua camera (Qimaging) and the Volocity software (Perkin Elmer).

In some cases the cell walls of sectioned algal materials were treated with enzymes prior to antibody labelling. The microscopic slides were coated with 1% poly-L-lysine (Sigma) and the washed sections were allowed to dry on the slides. Removal of alginates was performed using a mixture of 5 µg/mL of the alginate lyase AlyA1 from *Zobellia galactinovorans* ^30^ and 5 µg/mL of an alginate lyase from *Pseudomonas alginovora* ^29^, in 25 mM Tris pH7.5, 200 mM NaCl for 1 h at room temperature. Degradation of MLG was performed using the lichenase from *Bacillus sp*. (Megazyme) at a concentration of 10 µg/mL in 100 mM Tris pH6.5, 200 mM NaCl for 1 h at room temperature. Sections not treated with the enzymes were incubated for an equivalent time with the corresponding buffers.

## Electronic supplementary material

Supplementary Information
