# Supplementary material for: Insoluble (1 → 3), (1 → 4)-β-D-glucan is a component of cell walls in brown algae (Phaeophyceae) and is masked by alginates in tissues
Source: Sci Rep. 2017 Jun 6;7:2880. doi: 10.1038/s41598-017-03081-5 (PMC5460208; doi:10.1038/s41598-017-03081-5)
Supplement: Supplementary file 1 — Supplementary Information [file 41598_2017_3081_MOESM1_ESM.pdf]

Insoluble (1→3),(1→4)-β-D-glucan is a component of cell walls in brown algae (Phaeophyceae) and is masked by alginates in tissues

Armando A. Salmeán[1], Delphine Duffieux[2,3], Jesper Harholt[4], Fen Qin[4], Gurvan Michel[2,3], Mirjam Czjzek[2,3], William G.T. Willats[1,5,*] and Cécile Herve[2,3,*]

[1]*Department of Plant and Environmental Sciences, University of Copenhagen, Thorvaldsensvej 40, 1871 Frederiksberg, Denmark;* [2]*Sorbonne Universités, UPMC Univ Paris 06, UMR 8227, Integrative Biology of Marine Models, Station Biologique de Roscoff, CS 90074, Roscoff, France;* [3]*CNRS, UMR 8227, Integrative Biology of Marine Models, Station Biologique de Roscoff, CS 90074, Roscoff, France;* [4]*Carlsberg Research Laboratory, J.C. Jacobsens Gade 4, 1799 København V, Denmark*

[5]*Present address: William G.T. Willats, Newcastle University, Newcastle upon Tyne, United Kingdom*

[*]*Correspondence: William G.T. Willats, william.willats@newcastle.ac.uk; Cécile Hervé, cecile.herve@sb-roscoff.fr*

	(1→3),(1→4)-β-D-glucan (mAb BS-400-3)					alginates (mAb LM7)				
EXTRACTS:	$CaCl_2$	HCl	Na_2CO_3	CDTA	NaOH	$CaCl_2$	HCl	Na_2CO_3	CDTA	NaOH
FUCALES										
Pelvetia canaliculata	0	0	0	0	22	0	0	43	7	0
Fucus distichus	0	0	0	0	32	0	0	57	30	0
Fucus serratus	0	0	0	0	33	0	0	56	38	0
Fucus spiralis	0	0	0	6	30	0	0	73	18	0
Fucus vesiculosus	0	0	0	0	32	0	0	46	50	0
Ascophyllum nodosum	0	0	0	0	28	0	0	61	24	0
Durvillaea antartica	0	0	0	0	27	0	0	88	41	9
Durvillaea potatorum	0	0	0	0	26	0	0	52	52	6
Himanthalia elongata	0	0	0	0	39	0	0	76	13	0
Cystoseira nodicaulis	0	0	0	0	48	0	0	48	50	0
Sargassum tenerrimum	0	0	0	0	44	0	0	84	0	0
Sargassum wightii	0	0	0	0	38	0	0	80	0	0
Sargassum longifolium	0	0	0	0	48	0	0	100	21	9
Sargassum muticum (blade)	0	0	0	0	42	0	0	77	14	0
Sargassum muticum (stipe)	0	0	0	0	43	0	0	47	16	0
Bifurcaria bifurcata	0	0	0	0	44	0	0	57	38	0
TILOPTERIDIALES										
Saccorhiza polyschides (stipe)	0	0	0	0	30	0	0	67	23	0
Saccorhiza polyschides (holdfast)	0	0	0	0	30	0	0	35	45	6
LAMINARIALES										
Laminaria nigripes (blade)	0	0	0	0	33	0	0	41	62	10
Laminaria nigripes (stipe)	0	0	0	0	30	0	0	58	54	9
Laminaria ochroleuca (blade)	0	0	0	0	43	0	0	53	30	0
Laminaria ochroleuca (holdfast)	0	0	0	0	44	0	0	63	64	9
Sacharina latissima (blade)	0	0	0	0	36	0	0	85	33	0
Sacharina latissima (stipe)	0	0	0	0	39	0	0	76	23	0
Sacharina latissima (holdfast)	0	0	0	0	28	0	0	34	6	0
Saccharina japonica	0	0	0	0	39	0	0	84	89	13
Laminaria digitata (blade)	0	0	0	0	33	0	0	81	61	8
Laminaria digitata (stipe)	0	0	0	0	46	0	0	65	82	12
Laminaria digitata (holdfast)	0	0	0	0	30	0	0	52	21	0
Laminaria hyperborea (blade)	0	0	0	0	45	0	0	88	26	0
Laminaria hyperborea (meristem)	0	0	0	0	52	0	0	71	69	7
Laminaria hyperborea (stipe)	0	0	0	0	40	0	0	77	47	7
Laminaria hyperborea (holdfast)	0	0	0	0	33	0	0	51	36	0
Macrocystis pyrifera (blade)	0	0	0	0	46	0	0	62	57	0
Macrocystis pyrifera (stipe)	0	0	0	0	41	0	0	54	46	0
Agarum clathratum (blade)	0	0	0	0	40	0	0	35	20	0
Agarum clathratum (stipe)	0	0	0	0	28	0	0	70	59	8
Alaria esculenta (blade)	0	0	0	0	30	0	0	58	15	0
Alaria esculenta (stipe)	0	0	0	0	37	0	0	63	27	0
Undaria pinnatifida (blade)	0	0	0	0	50	0	0	67	62	0
Undaria pinnatifida (stipe -upper part)	0	0	0	15	46	0	0	77	58	10
Undaria pinnatifida (stipe -lower part)	0	0	0	0	36	0	0	63	86	11
Ecklonia maxima	0	0	0	0	44	0	0	55	48	0
Eisenia bycyclis	0	0	0	0	45	0	0	42	0	0
Lessonia nigrescens (blade)	0	0	0	0	51	0	0	69	24	0
Lessonia nigrescens (holdfast)	0	0	0	0	27	0	0	26	16	0
Lessonia trabeculata (blade)	0	0	0	0	46	0	0	100	18	10
Lessonia trabeculata (holdfast)	0	0	0	0	32	0	0	60	20	7
ECTOCARPALES										
Pylaiella littoralis	0	0	0	0	46	0	0	24	0	0
Ectocarpus siliculosus	0	0	0	0	44	0	0	68	0	0
Colpomenia peregrina	0	0	0	0	44	0	0	45	0	0
SPHACELARIALES										
Stypocaulon scoparium	0	0	0	0	55	0	0	68	0	0
DICTYOTALES										
Dictyota dichotoma	0	0	0	0	42	0	0	86	10	0
Dictyopteris membranacea	0	0	0	0	25	0	0	39	0	0
Relative mean signal:	0-10	11-20	21-40	41-60	61-100					

Supplementary Figure S1. Full dataset showing the detection of MLG epitopes in brown algae among the different cell wall extracts and tissue-types. The heatmap was obtained as explained for Fig. 1.

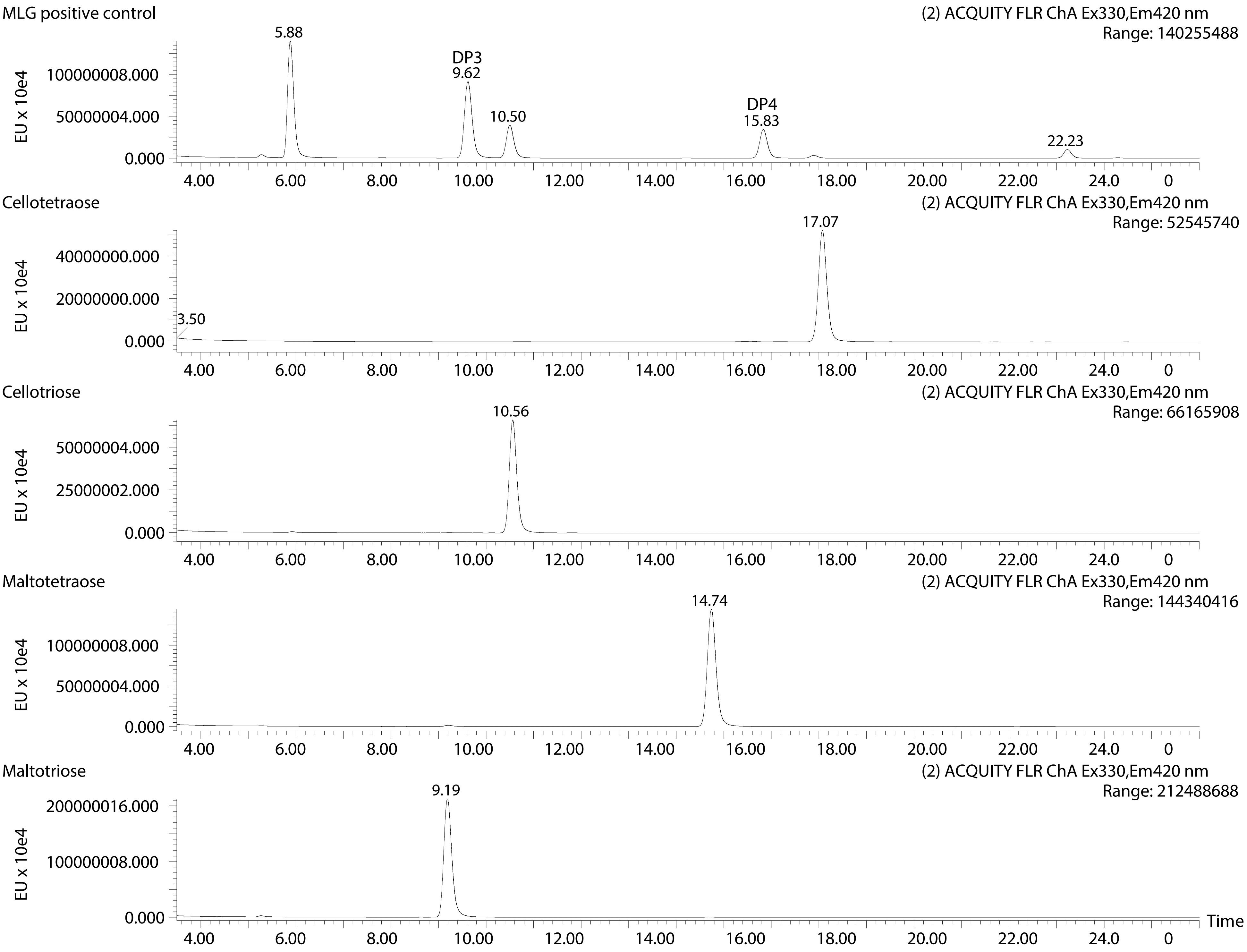
MLG positive control
(2) ACQUITY FLR ChA Ex330,Em420 nm
Range: 140255488
EU x 10e4
100000008.000
50000004.000
0.000
5.88
DP3
9.62
10.50
DP4
15.83
22.23
Cellotetraose
(2) ACQUITY FLR ChA Ex330,Em420 nm
Range: 52545740
40000000.000
20000000.000
3.50
17.07
Cellotriose
(2) ACQUITY FLR ChA Ex330,Em420 nm
Range: 66165908
50000004.000
25000002.000
10.56
Maltotetraose
(2) ACQUITY FLR ChA Ex330,Em420 nm
Range: 144340416
14.74
Maltotriose
(2) ACQUITY FLR ChA Ex330,Em420 nm
Range: 212488688
200000016.000
100000008.000
9.19
Time
4.00 6.00 8.00 10.00 12.00 14.00 16.00 18.00 20.00 22.00 24.0 0

Supplementary Figure S2. Ultra-Performance Liquid Chromatography (UPLC) analysis of Glucagel, cellotetraose, cellotriose, maltotetraose and maltotriose. They were analysed as described in the methods. There was no co-elution between the MLG trisaccharide or any of the standards tested. The Glucagel contain some impurities giving rise to non-MLG peaks such as the peak at retention time 10.50 minute.

(a)

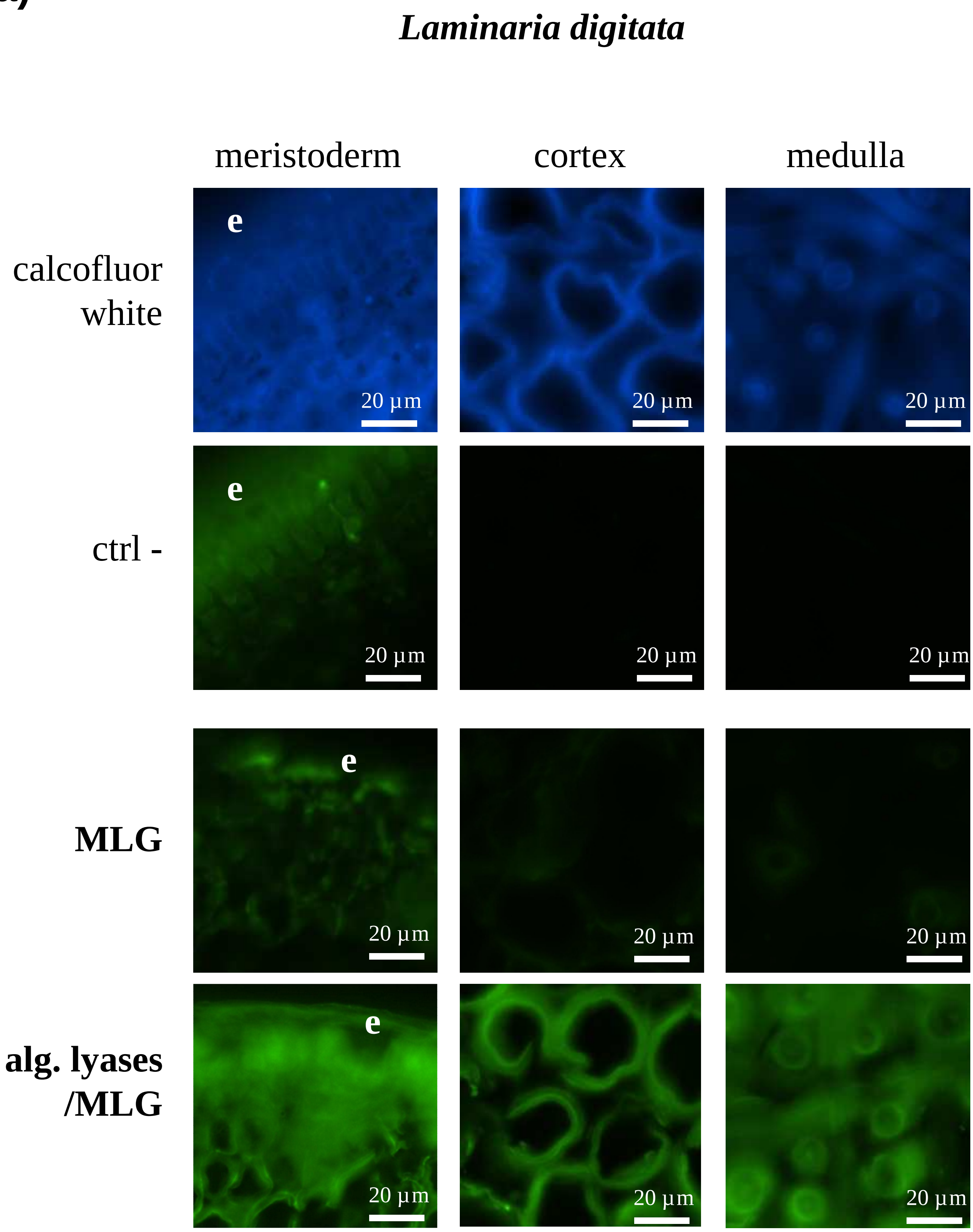
Laminaria digitata
meristoderm
cortex
medulla
calcofluor white
ctrl -
MLG
alg. lyases /MLG
e
20 µm

(b)

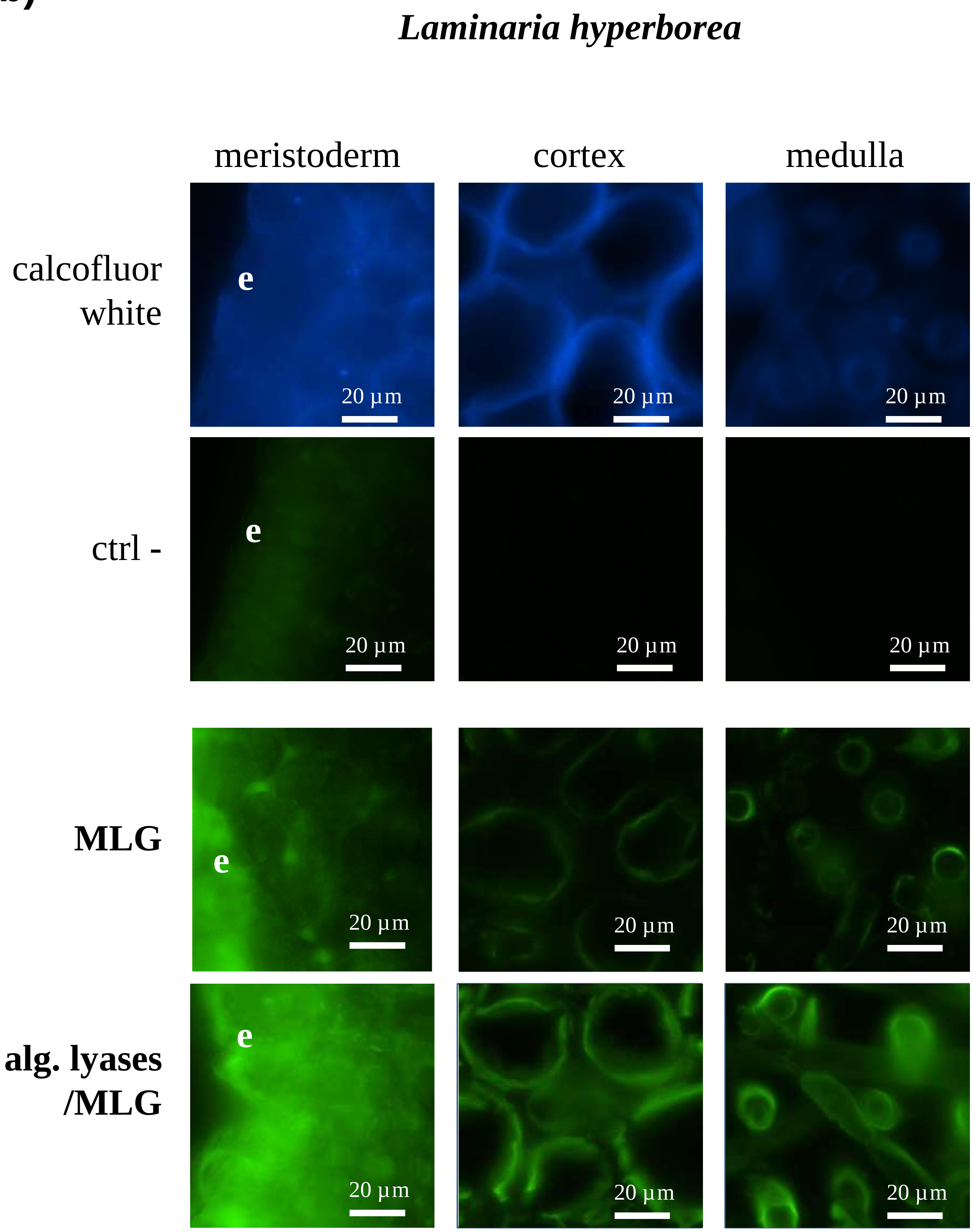
Laminaria hyperborea
meristoderm
cortex
medulla
calcofluor white
ctrl -
MLG
alg. lyases /MLG
e
20 µm

(c)

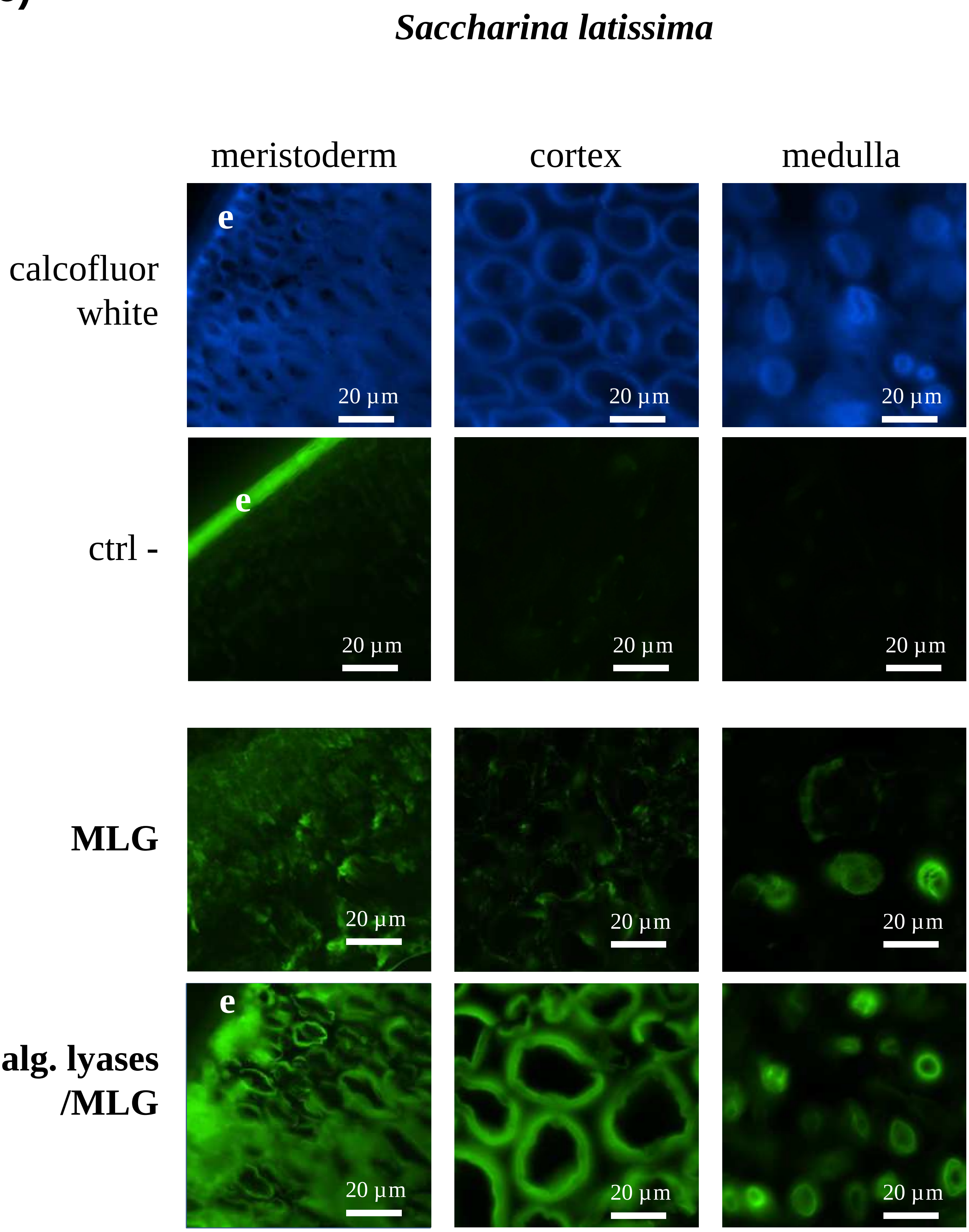
Saccharina latissima
meristoderm
cortex
medulla
calcofluor white
ctrl -
MLG
alg. lyases /MLG
e
20 µm

Supplementary Figure S3. MLG detection in stipes from Laminariales by immunofluorescence imaging. The micrographs show the indirect immunofluorescence detection of MLG epitopes with and without the alginate lyase pre-treatment in (**a**) *Laminaria digitata*, (**b**) *Laminaria hyperborea* and (**c**) *Saccharina latissima*. Equivalent sections stained with Calcofluor White and labelled with the secondary antibody only (negative control) are shown for comparison and indicate all cell walls in sections and autofluorescence of tissues, respectively. Note that the epidermis shows strong autofluorescence in most cases, rendering it difficult to observe the binding in the meristoderm located below. Some MLG epitopes are detected without the enzymatic degradation of alginates, but are more strongly apparent after the treatment. All scale bars = 20 μm. e =epidermis
